# Vaping cessation interventions: A narrative review of randomized controlled trials

**DOI:** 10.18332/tpc/222678

**Published:** 2026-07-23

**Authors:** Tetiana Zolotarova, Areesha Moiz, Audrey Lelièvre, Mark J. Eisenberg

**Affiliations:** 1 Centre for Clinical Epidemiology, Lady Davis Institute, Jewish General Hospital/McGill UniversityMontrealCanada; 2 Department of Epidemiology, Biostatistics and Occupational HealthMcGill UniversityMontrealCanada; 3 Department of MedicineMcGill UniversityMontrealCanada; 4 Division of Cardiology, Jewish General Hospital/McGill UniversityMontrealCanada

**Keywords:** vaping cessation, e-cigarettes, young adults, behavioral interventions, pharmacotherapy

## Abstract

The increasing prevalence of electronic cigarette (e-cigarette) use, particularly among young individuals, has led to a pressing need for effective vaping cessation interventions. We conducted a narrative review of published and ongoing randomized controlled trials (RCTs) evaluating behavioral, pharmacological, and combination interventions aimed at vaping cessation among adolescents and adults using e-cigarettes. PubMed, Google Scholar, and ClinicalTrials.gov were searched from 2004 (year of first e-cigarette patent) to 27 April 2025. The outcome of interest was abstinence from vaping at the maximum follow-up and results were synthesized narratively. A total of 11 RCTs, involving 8182 participants, were identified. These trials investigated behavioral therapy, including tailored text messaging support (n=4), nicotine replacement therapy (NRT) (n=3), varenicline (n=3), and cytisine (n=1). Studies were published between 2021 and 2025, with follow-up ranging from 1 to 24 months, and most focused on adults or young adults. Behavioral interventions, particularly text messaging programs, showed potential to support vaping cessation beyond 6 months, although findings were inconsistent across trials. Varenicline demonstrated promise as a pharmacological aid; however, available RCTs had small sample sizes and short follow-up periods, limiting the strength of the evidence. None of the RCTs investigating the efficacy of NRT (567 participants) or cytisine (160 participants) demonstrated superiority over control following treatment discontinuation. Fourteen ongoing RCTs were also identified, most of which are evaluating behavioral approaches in younger populations. Overall, the current evidence base for vaping cessation remains limited, highlighting the need for larger, high-quality trials with longer follow-up to establish effective interventions across diverse populations.

## Introduction

The use of electronic cigarettes (e-cigarettes), or ‘vaping’, has increased in recent years, with over 14 million adults in the United States now reporting daily use^1^. Use is highest among young adults aged 18–24 years, >65% of whom have never smoked combustible cigarettes^1^. In contrast, among individuals aged ≥65, over 95% who vape are current or former cigarette smokers^1^. The US e-cigarette market has reached a value of $12.4 billion in 2023, with global projections expected to hit $77.9 billion by 2030^2^. Despite the increasing use of e-cigarettes, over half of individuals who vape daily report a desire to quit^3^. Behavioral and pharmacological interventions commonly used for conventional smoking cessation, including text-based counseling programs, nicotine replacement therapy (NRT), varenicline, and cytisine, are increasingly being explored for vaping cessation; however, evidence supporting their efficacy remains limited and heterogeneous^4^. This narrative review aims to summarize the strengths and limitations of individual trials on vaping cessation interventions while also offering an overview of ongoing studies to provide a comprehensive and up-to-date assessment of the available evidence.

We conducted a narrative review of published and ongoing randomized controlled trials (RCTs) evaluating vaping cessation interventions among individuals using e-cigarettes (or other vaping devices) daily. The databases searched to identify trials were PubMed, Google Scholar, and ClinicalTrials.gov. The search period ranged from January 2004 (the year of the first e-cigarette patent in any language) to 27 April 2025. Medical subject heading terms were used when relevant for the following key words: ‘vaping cessation’, ‘e-cigarette cessation’, ‘electronic cigarettes’, ‘electronic nicotine delivery systems’, ‘intervention’, and ‘randomized controlled trial’. Relevant search results were identified through title screening or snowballing references of included articles. Studies were reviewed for relevance based on study design, population, and intervention characteristics. The outcome of interest was abstinence from vaping at the maximum follow-up.

Eligible studies included RCTs published in English evaluating behavioral, pharmacological, or combination interventions for vaping cessation among adolescents or adults using e-cigarettes. Study screening and data extraction were conducted manually by one reviewer (TZ). Extracted variables included study population characteristics, intervention type, sample size, abstinence outcome definitions, effect estimates and corresponding variance measures, biochemical verification methods, and maximum follow-up duration. Findings were synthesized narratively, given the heterogeneity in study populations, interventions, and outcome definitions.

## Developments

We identified 11 published RCTs published between 2021 and 2025, evaluating a range of vaping cessation interventions. These included four trials of behavioral therapies^5-8^, three NRT trials^9-11^ and four pharmacotherapy trials – three with varenicline^12-14^ and one with cytisine^15^. Collectively, the studies enrolled 8182 participants, with follow-up durations ranging from 1 to 24 months (Tables 1–3). Most trials targeted adults or young adults (age 18–25 years)^5-7,9-15^, with one trial exclusively enrolling adolescents^8^. The most frequently reported primary outcome was 7 day point-prevalence abstinence (PPA) from vaping, defined as self-reported nicotine e-cigarette abstinence within the past 7 days. Four studies incorporated biochemical verification using salivary cotinine testing, with cutoff thresholds ranging from 10 to 30 ng/mL^7,12,13,15^.

**Table 1:** Characteristics of published randomized controlled trials evaluating behavioral interventions for vaping cessation among adolescents and young adults, 2021–2024

Study	Sample size	Inclusion criteria	Interventions	Outcome measure	Biochemical validation of abstinence	Maximum follow-up (months)
Martinez et al.^5^ 2021	2896	Age ≥18 years Current 30 day EC use Combustible cigarettes use	Cessation materials (booklets, links) vs Cessation materials designed for dual usersvs Control*	7 day PPA	Exhaled CO (<8 ppm) Saliva cotinine test (who lived <100 miles from the research site, n=47)	24
Graham et al.^6^ 2021	2588	Age 18–24 years Current 30 day EC use	Text messaging support vs Control*	30 day PPA	-	7
Palmer et al.^7^ 2022	27	Age 12–21 years Current (>25 days/month) EC use	Financial incentives vs Control*	Vaping abstinence	Saliva cotinine test (<30 ng/mL^−1^)	1
**Adolescents only**						
Graham et al.^8^ 2024	1503	Age 13–17 years Current 30 day EC use	Text messaging support vs Control*	30 day PPA	-	7

Studies were identified through a literature search conducted from January 2004 to 27 April 2025.

* The control group did not receive any active intervention and, at most, was referred to publicly available self-guided behavioral resources.

CO: carbon monoxide; EC: electronic cigarette; PPA: point prevalence abstinence.

**Table 2:** Characteristics of published randomized controlled trials evaluating nicotine replacement therapy for vaping cessation among young adults, 2021–2025

Study	Sample size	Inclusion criteria	Interventions	Outcome measure	Biochemical validation of abstinence	Maximum follow-up (months)
Vickerman et al.^9^ 2025	508	Age 18–24 years Current 30 day EC use	Coaching+Text messaging support+NRT^a^ vs Coaching+NRT^a^ vs Coaching+Text messaging support vs Coaching only	7 day PPA	-	3
Palmer et al.^10^ 2023	30	Age ≥18 years Current (>25 days/month) EC use Dual users were permitted	NRT^b^ vs Control*	7 day PPA	-	1
Sahr et al.^11^ 2021	29	Age 18–24 years Current (≥16 days/months) EC use	NRT^c^ vs Vape-tapervs Control*	Self-report being vape-free	-	6

Studies were identified through a literature search conducted from January 2004 to 27 April 2025.

a NRT: up to an 8-week supply of nicotine patch, gum, and/or lozenge.

b NRT was supplied for 28 days.

c NRT was supplied for 12 weeks.

* The control group did not receive any active intervention and, at most, was referred to publicly available self-guided behavioral resources.

EC: electronic cigarette; NRT: nicotine replacement therapy; PPA: point prevalence abstinence.

**Table 3:** Characteristics of published randomized controlled trials evaluating varenicline and cytisine for vaping cessation among young adults, 2023–2025

Study	Sample size	Inclusion criteria	Interventions	Outcome measure	Biochemical validation of abstinence	Maximum follow-up (months)
**Varenicline**						
Evins et al.^12^ 2025	261	Age 16–25 years Daily EC use	Varenicline^a^ vs Placebo^a^ vs Text messaging support	CAR (weeks 9–24)	Saliva cotinine test (cutoff <30 ng/mL^−1^)	6
Caponetto et al.^13^ 2023	140	Age ≥18 years Daily EC use	Varenicline^a^ vs Placebo^a^	CAR (weeks 4–24) 7 day PPA	Saliva cotinine test (cutoff <10 ng/mL^−1^)	6
Fucito et al.^14^ 2024	40	Age ≥18 years Daily EC use >6 months	Varenicline^b^ vs Placebo^b^	7 day PPA	-	3
**Cytisine**						
Rigotti et al.^15^ 2024*	160	Age ≥18 years Daily EC use Positive (≥30 ng/mL^−1^) saliva cotinine test result	**Cytisinicline**^a^ **vs Placebo**^a^	CAR (weeks 9–12; 9–16) 7 day PPA	Saliva cotinine test (cut-off <10 ng/mL^−1^)	4

Studies were identified through a literature search conducted from January 2004 to 27 April 2025

a Supplied for 12 weeks.

b Supplied for 8 weeks.

* The results were reported via a figure; no numerical values were provided

CAR: continuous abstinence rate; EC: electronic cigarette; PPA: point prevalence abstinence.

### Adults

#### Behavioral interventions

We identified three RCTs (n=5511 participants) investigating the effects of behavioral interventions for e-cigarette cessation (Table 1)^5-7^. The trials varied in population and intervention delivery approaches.

Martinez et al.^5^ conducted the largest RCT (n=2896 participants) in vaping cessation using behavioral intervention, which included three study arms: one control group and two self-help intervention groups. One intervention arm received targeted cessation materials (brochures, booklets, etc) designed specifically for individuals who exclusively use e-cigarettes, while the other received materials tailored for dual users – individuals who smoke both conventional cigarettes and nicotine e-cigarettes. Both intervention arms received self-guided behavioral therapy materials by mail for up to 18 months. As part of the retention strategy, participants received $10 to $40 for follow-up assessments, with additional bonuses and appreciation gifts for timely and consistent participation. At 24 months, no significant differences were observed based on 7 day PPA from vaping across groups (Odd ratios [OR]=1.07; 95 Confidence Interval [CI]: 0.82–1.41 and OR=1.17; 95% CI: 0.88–1.54, respectively) (Table 4). Methodological limitations included substantial loss to follow-up (53% at 24 months) despite the imputation approach. While biochemical validation was conducted, it was limited to a small subset of participants (n=47) living within 100 miles of the research site, leaving most abstinence outcomes based on self-report. The intervention largely relied on self-guided cognitive behavioral therapy delivered via printed materials with no in-person or digital support.

**Table 4:** Reported abstinence outcomes of published randomized controlled trials evaluating behavioral and pharmacological vaping cessation interventions among adolescents and young adults

Interventions	Sample size	7 day or 30-day PPA from vaping at maximum follow-up (%)	Effect estimates and significance (95% CI)	Continuous abstinence from vaping (%)	Effect estimates and significance (95% CI)
**Behavioral interventions**					
**Martinez et al.**^**5**^ **(2021)**					
Cessation materials (booklets, links)	1154	34.0	OR=1.07 (0.82–1.41)**	NA	NA
Cessation materials designed for dual users	1167	35.9	OR=1.17 (0.88–1.54)**		
Control*	575	32.4	NA		
**Graham et al.**^**6**^ **(2021)**					
Text messaging support	1304	24.1	OR=1.17 (0.88–1.54) RR=1.35 (1.17–1.57)	NA	NA
Control*	1284	18.6			
**Palmer et al.**^**10**^ **(2022)**					
Financial incentives	22	27.0	p=0.74	NA	NA
Control*	55	20.0			
**Graham et al.**^**8**^ **(2024)**					
Text messaging support	759	38.7	OR=1.57 (1.26–1.95) RR=1.35 (1.17–1.57)	NA	NA
Control*	744	28.0			
NRT					
**Vickerman et al.**^9^ **(2025)**					
Coaching+Text messaging support+NRT	122	48.0	NRT arms vs non-NRT arms OR=1.30 (0.91–1.84)	NA	NA
Coaching+NRT	126	48.0			
Coaching+Text-messaging support	126	43.0			
Coaching only	134	41.0			
**Palmer et al.**^**10**^ **(2023)**					
NRT	18	27.8	p=0.67	NA	NA
Control*	12	16.7			
**Sahr et al.**^**11**^ **(2021)**					
NRT	7	42.9	p=0.35	28.6	p=0.44
Vape-taper	8	75.0		37.5	
Control*	9	44.4		11.5	
**Varenicline**					
**Evins et al.**^**12**^ **(2025)**					
Varenicline	88	NA	NA	28	AOR=6.0 (2.1–16.9)^a^
Placebo	87			7	
Text messaging support	86			4	AOR=11.0 (3.1–38.8)^b^
**Caponetto et al.**^**13**^ **(2023)**					
Varenicline	70	34.3	OR=2.52 (1.14–5.58) p=0.02	34.3	OR=2.52 (1.14–5.58) p=0.02
Placebo	70	17.1		17.1	
**Fucito et al.**^**14**^ **(2024)**					
Varenicline	20	40.0	RR=1.36 (0.59–3.13)	NA	NA
Placebo	20	30.0			
**Cytisine**					
**Rigotti et al.**^**15**^ **(2024)**					
Cytisinicline	107	NA	NA	23.4	OR=2.00 (0.82–5.32)
Placebo	53	NA	NA	13.2	

Studies were identified through a literature search conducted from January 2004 to 27 April 2025.

AOR: adjusted odds ratio. CI: confidence interval. NA: not applicable. NRT: nicotine replacement therapy. PPA: point prevalence abstinence. RR: relative risk.

a Varenicline vs placebo arm.

b Varenicline vs text messaging support arm

* The control group did not receive any active intervention and, at most, was referred to publicly available self-guided behavioral resources.

** Compared to the control arm.

Graham et al.^6^ conducted a parallel, two-group, double-blind RCT (n=2588) to evaluate the efficacy of *This is Quitting*, a fully automated, interactive text message program. The program was designed to support vaping cessation in young adults. A distinctive feature of the program was the inclusion of peer-contributed messages from other individuals who vape. The intervention group received daily supportive and skill-building messages for up to 9 weeks. All participants received incentivized text message assessments about e-cigarette use and abstinence at 14 days post-randomization and monthly for 6 months. The control group did not receive any additional support. Participants received up to $35 for follow-up assessments, $20 for each follow-up survey, and a $10 bonus for responding within 24 hours. At the 7 month follow-up, a higher proportion of participants in the intervention group reported 30 day vaping abstinence compared to the control group (24.1% vs 18.6%; Relative Risk [RR]=1.35; 95% CI: 1.17–1.57) (Table 4). The study had several strengths, including a large, diverse sample, high retention, and rigorous design. However, it relied on self-reported abstinence without biochemical verification, and recruitment was conducted online.

Palmer et al.^7^ conducted a pilot feasibility study evaluating the use of financial incentives, also known as contingency management, for vaping cessation. Participants (n=27) were randomized in a 4 : 1 ratio to intervention or a control group. Participants in the intervention group received $20 for each submitted sample that was biochemically verified as abstinent. The control group received $20 for each submitted sample, regardless of the result. Participants could earn up to $310, including abstinence or submission incentives ($200), pre-quit saliva submissions ($15), and compensation for study visits ($95). The intervention was delivered via a smartphone app over a 4 week period. All participants were encouraged to use the *This is Quitting* text message program. No significant difference in vaping abstinence at 56 days was found between the financial incentive and the control groups (27.0% vs 20.0%; p=0.74) (Table 4). Although participants in the intervention arm submitted a higher proportion of negative samples during treatment (55% vs 8%), the study was limited by its small sample size as well as its short-term intervention and follow-up periods.

#### Nicotine replacement therapy

Three RCTs (n=567 participants) assessed the efficacy of NRT for nicotine e-cigarette cessation (Table 2)^9-11^. All studies combined NRT with behavioral interventions, with treatment durations ranging from 28 days to 12 weeks.

Vickerman et al.^9^ conducted an RCT (n=508) to evaluate the efficacy of NRT and a text-messaging program for vaping cessation among young adults aged 18–24 years. A 2×2 factorial design was used to create the study groups: 1) coaching calls + text-messaging support + NRT, 2) coaching calls + NRT, 3) coaching calls + text-messaging support, and 4) coaching calls only. NRT was delivered by mail and included an 8 week supply of nicotine patches and gum or lozenges. All participants received $40 electronic gift cards for baseline and 3 month survey completion. At the follow-up at 3 months, 45% of participants reported vaping abstinence, with NRT associated with a 6.5% increase in abstinence compared to non-NRT groups, though the effect was inconclusive (OR=1.30; 95% CI: 0.91–1.84) (Table 4). The study had several strengths, including multisite recruitment, a relatively large sample for vaping cessation research, and a fully remote delivery model. However, the abstinence was self-reported, and the follow-up period was short-term. Notably, NRT was provided for only 8 weeks, whereas most conventional smoking cessation trials use a 12 week course. NRT use was also reported among participants not assigned to receive it. In addition, the sample had a higher proportion of female participants (71.3%).

Palmer et al.^10^ conducted a mixed-methods preliminary study (n=30) to evaluate the feasibility and acceptability of NRT for vaping cessation among adults who exclusively used e-cigarettes and dual users. Participants were randomized to receive either a 28 day supply of combination NRT (patches and lozenges) or a referral to telephone-based cessation services (control group). All participants received $20 per assessment (up to $60), while those randomized to the intervention group could earn an additional $60 for completing daily surveys. All payments were electronic gift cards. At follow-up at 1 month, among participants who exclusively used e-cigarettes, one-third in the NRT group reported vaping abstinence, compared to none in the control group. Overall, abstinence rates in the NRT group (33.3%) showed a trend toward being higher than in the control group (0%), though the difference was inconclusive (p=0.67) (Table 4). The main limitations were small sample size, short follow-up, and reliance on self-reported abstinence without biochemical validation. Moreover, NRT therapy was limited to 28 days, and delivered in fixed doses rather than tailored to individual dependence levels.

Sahr et al.^11^ conducted a pilot RCT (n=24) comparing three vaping cessation methods: 1) NRT, 2) vape-taper, and 3) control (self-guided vaping cessation). Individuals in the vape-taper arm were encouraged to reduce nicotine intake by lowering the vape liquid concentration and decreasing session frequency or duration, aiming to eliminate one session daily. Participants received a $20 gift card at follow-up visits, with a maximum of $60. Those in the self-guided and vape-taper groups received an additional $120 to cover vaping supply costs. At 6 months, the vape-taper group had the highest self-reported abstinence rates (75.0%), followed by the self-guided (44.4%) and NRT groups (42.9%) (Table 4). However, the trial was underpowered and had limited generalizability, as all participants were college students from a single campus, introducing potential sampling bias. A high number of participants were lost to follow-up, particularly in the NRT group, which may have impacted the reliability of outcomes. Additionally, many individuals in the NRT arm used the products at lower intensity than recommended.

#### Varenicline

We identified three trials (n=441 participants) evaluating the efficacy and safety of varenicline for vaping cessation in the general population (Table 3)^12-14^.

Evins et al.^12^ conducted a three-arm, double-blind, placebo-controlled RCT (n=261) to evaluate the efficacy of varenicline for vaping cessation among youth aged 16–25 years. Participants were randomized to receive 12 weeks of varenicline or placebo with weekly counseling, or to enhanced usual care (text message support via *This is Quitting*). Participants received up to $570 for completing assessments, with those in the varenicline and placebo arms additionally incentivized $1 per video-confirmed medication dose via a reminder app. At follow-up at 6 months, the varenicline arm had a higher continuous abstinence rate compared to placebo (Adjusted Odds Ratio [AOR]=6.0; 95% CI: 2.1–16.9) (Table 4). It also outperformed enhanced usual care (AOR=11.0; 95% CI: 3.1–38.8). Adverse events were mild and similar between groups. The trial’s strengths include its rigorous design, high adherence and retention, and robust biochemical verification. However, limitations include a single-site design and a lack of generalizability to dual users.

Caponnetto et al.^13^ conducted a double-blind, placebo-controlled RCT (n=140) to evaluate the efficacy of varenicline combined with behavioral counseling for vaping cessation among adults who exclusively used e-cigarettes. Participants received varenicline or a placebo for 12 weeks alongside structured counseling. Both groups also received instructions to gradually reduce their vaping product use at their own pace as an additional intervention. At follow-up at 6 months, varenicline more than doubled the odds of biochemically verified abstinence from vaping compared to placebo (OR=2.52; 95% CI: 1.14–5.58) (Table 4). The continuous vaping abstinence indicated similar results. The key limitations included a relatively small sample size and short-term follow-up. The study was funded by Pfizer Inc. (USA) and partially supported by ECLAT Srl., a research-based company specializing in combustion-free devices. However, the investigators stated that neither funder had any influence on the study design or conduct.

Fucito et al.^14^ reported the results of a double-blind, placebo-controlled RCT evaluating the effect of an 8 week varenicline regimen among 40 enrolled participants. All participants received a single low-intensity counseling session and a self-guided booklet. At 3 months, the differences in abstinence rates between the varenicline and placebo groups were inconclusive (40.0% vs 30.0%; RR=1.36; 95% CI: 0.59–3.13) (Table 4). Strengths of the study included its primary care-based setting and inclusion of participants with psychiatric comorbidities, reflecting a real-world population. However, the study was underpowered, with a short follow-up and a lack of biochemical verification. Notably, the varenicline treatment course was shorter than a standard 12 week regimen used in smoking cessation.

#### Cytisine

Rigotti et al.^15^ conducted a double-blind, placebo-controlled RCT (n=160) to evaluate the efficacy of cytisinicline for vaping cessation in adults who exclusively used nicotine e-cigarettes. Participants followed a simplified dosing regimen of 3 mg cytisinicline, taken 3 times daily for 12 weeks, combined with weekly brief behavioral counseling (Table 3). Study participants were compensated for their time spent attending study visits, but no further details were provided by the authors. Cytisinicline more than doubled the likelihood of biochemically validated continuous abstinence compared to placebo from week 9 to 12. However, the effect was inconclusive after medication discontinuation (OR=2.00; 95% CI: 0.82–5.32) (Table 4). Although the study reported 7 day PPA from vaping at 4 months, no numerical values were provided, limiting the ability to compare this outcome across RCTs. The study was partially funded by Achieve Life Sciences, a pharmaceutical company that developed and commercializes cytisinicline for smoking cessation and nicotine addiction.

### Adolescents

We identified a single RCT (n=1503) on vaping cessation that evaluated the efficacy of a behavioral intervention exclusively among adolescents. Graham et al.^8^ conducted a parallel, two-group, double-blind RCT comparing text messaging support through *This is Quitting* to a control. Participants were recruited via social media advertisements (Instagram, Facebook, and Snapchat). To optimize follow-up rates, all participants received $5 for completing text message assessments about e-cigarette use monthly for 6 months. At 7 months, a higher proportion of participants in the intervention group reported 30 day vaping abstinence compared to the control group (38.7% vs 28.0%; RR=1.35; 95% CI: 1.17–1.57) (Table 4). The study’s strengths included its large sample, diverse population, and rigorous design. However, reliance on self-reported abstinence without biochemical verification, along with a loss to follow-up rate exceeding 25%, may introduce misclassification bias.

### Ongoing clinical trials

Our search identified 14 ongoing RCTs at different stages of completion on the ClinicalTrials.gov database, assessing interventions for achieving abstinence from vaping (Table 5). Some are in the early stages of recruitment, while others have completed follow-up, but the results are pending. Two-thirds of these trials are investigating the effects of behavioral interventions, such as mobile vaping apps and virtual reality programs on vaping cessation. Half of the trials primarily target younger populations (age 13–21 years).

**Table 5:** Characteristics of ongoing, completed, and terminated randomized controlled trials evaluating vaping cessation interventions among adolescents and young adults

Title	RCT ID	Target sample size	Age (years)	Interventions	Main outcome	Status date
**Behavioral interventions**						
Kick-Nic! Youth Quit Vaping App	NCT06662305	306	13–19	Vaping cessation app vs Control	Cotinine-confirmed 7 day PPA	Ongoing 2029-08-31
Testing the Feasibility and Acceptability of Social Media and Digital Therapeutics to Decrease Vaping Behaviors	NCT05994209	189	15–25	Vaping cessation app+Chatbot feature vs Vaping cessation app vs Usual care	7 day PPA from vaping at 3 months	Ongoing 2025-03-01
Adolescent Inpatient Tobacco and Ends Intervention	NCT05936099	144	14–21	Counseling (cigarettes and EC focused) vs Control	Cotinine-confirmed 7 day PPA at 3 months	Ongoing 2025-12-20
Vaping Prevention and Vaping in Youth (Vapechat)	NCT06003439	119 (actual)	13–19	Virtual reality program vs Control	Past 30-day- and 7 day vaping frequency	Completed 2024-06-07
Goal2quitvaping For Nicotine Vaping Cessation Among Adolescents	NCT04951193	106 (actual)	16–20	Behavioral activation therapy app vs Usual care	7 day vaping PPA	Completed 2023-06-21
Quit Nicotine: E-Cig Cessation Intervention	NCT04898075	100	13–20	Financial incentives vs Control	Cotinine-confirmed change in 7 day PPA at 6 and 12 months	Ongoing 2025-01-05
Incentive-Based and Media Literacy-Informed Approaches to Improve Vaping Cessation	NCT05586308	80	19–29	Financial incentive+Text-messaging support+E-learning lessons vs Financial incentive+Text-messaging support vs Text-messaging support+E-learning lessons vs Text-messaging support	Cotinine-confirmed 7 day PPA at 3 months	Ongoing 2025-06-01
A Smartphone Application (Act on Vaping) For Vaping Cessation in Young Adults	NCT05897242	61 (actual)	18–30	Vaping cessation app vs Text-messaging support vs Control	Cotinine-confirmed 30 day PPA	Completed 2024-04-26
Vape-Free Text-Messaging: Pilot Study	NCT05906082	50	18–24	Text-messaging support vs Usual care	Cotinine-confirmed 7 day PPA at 1 month	Ongoing 2024-12-31
Adapting An Intervention for Vaping in Young Veterans	NCT06196489	20	18–30	Vaping cessation video vs Counseling	7 day PPA at 30 days	Ongoing 2024-06-01
**Pharmacotherapy**						
The Avenues Study: Dual Use Cessation	NCT06474299	500	>21	Varenicline+Counseling (1 or 4 sessions) (cigarettes and EC cessation focused) vs Varenicline+Counseling (1 or 4 sessions) (Cigarette cessation-focused) vs NRT+Counseling (1 or 4 sessions) (cigarettes and EC cessation focused) vs NRT+Counseling (1 or 4 sessions) (Cigarette cessation-focused)	ECO-confirmed 7 day PPA from cigarettes at 52 weeks Cotinine-confirmed 7 day PPA from vaping at 52 weeks	Ongoing 2028-07-01
Vaping Cessation Using the Ottawa Model for Smoking Cessation Among E-Cigarette Users (VC-OMSC)	NCT06164678	180	>18	Counseling+/-NRT vs Usual care	Continuous abstinence at 6- and 12 month follow-ups	Ongoing 2025-12-31
Concurrent vs. Sequential Cessation of Dual Cigarette and E-Cigarette Use	NCT06027840	40	>18	Varenicline (12 weeks) + Counseling+Cessation materials (concurrent cessation of cigarettes and EC) vs Varenicline (12 weeks) + Counseling+Cessation materials (cessation of cigarettes followed sequentially by cessation of e-cigarettes)	Cotinine-confirmed 7 day PPA at 12 weeks	Ongoing 2025-06-01
Varenicline for Nicotine Vaping Cessation in Non-Smoker Vaper Adolescents (Pilot)	NCT04602494	5 (actual)	18–25	Varenicline (12 weeks) vs Placebo vs Control	Continuous nicotine vaping abstinence from week 9 to 12	Terminated 2022-06-27

EC: electronic cigarette, ECO: exhaled carbon monoxide, NCT: National Clinical Trial identifier, PPA: point prevalence abstinence.

a Table includes ongoing, completed, and terminated randomized controlled trials registered on ClinicalTrials.gov for which published results were not identified at the time of the literature search on 27 April 2025.

One notable trial, the Avenue Study (NCT06474299)^16^, aims to identify the most effective strategies for helping dual users of cigarettes and e-cigarettes quit smoking. This RCT evaluates four arms, combining pharmacotherapy (varenicline or NRT) with behavioral counseling of varying intensity (1 or 4 sessions). The interventions either address both cigarette and e-cigarette cessation or focus solely on cigarette cessation. With a target enrollment of 500 participants, the study is expected to be completed in 2028. The primary outcome is 7 day PPA from cigarette smoking at 52 weeks, biochemically validated with exhaled carbon monoxide measurement (<6 ppm) for smoking abstinence. Abstinence from e-cigarette use will be verified using saliva cotinine measurement (<30 ng/mL^−1^).

Among the 11 published RCTs, behavioral interventions, particularly digital and text message-based programs, demonstrated potential to support vaping cessation among adolescents and young adults. Evidence for pharmacological interventions was more limited, although varenicline showed the most consistent signal of benefit across trials. In contrast, evidence supporting NRT and cytisine remains inconclusive because of the small number of available studies and relatively short follow-up durations. More than half of the trials were underpowered and did not include biochemical verification of vaping abstinence. Importantly, we also identified 14 ongoing RCTs evaluating behavioral and pharmacological interventions for vaping cessation, which may help address some of these current evidence limitations.

### E-cigarette safety concerns

While available evidence indicates that e-cigarette use may be less harmful than conventional tobacco smoking, this does not imply they are without risk. The potential long-term effects of e-cigarette use remain largely unexplored^17^. Most RCTs limit e-cigarette use for smoking cessation to a maximum of 14 weeks, with follow-up rarely extending beyond 1 year^4^. The 2025 Cochrane review concluded that safety data remain inconclusive^4^. Chronic nicotine exposure during adolescence is linked to cognitive deficits and increased risk of lifelong dependence^18,19^. Modern devices using nicotine salts deliver higher doses more efficiently, and their modifiability allows users to bypass regulatory limits in countries like Canada and United Kingdom^20,21^. A 2025 multinational study found that adolescents who vape had nicotine exposure levels comparable to those who smoke, with over one-third using the highest allowed concentration (20 mg ml^−1^)^22^. A separate meta-analysis of cross-sectional studies found that e-cigarette use was associated with a 1.5-fold increase in suicidal ideation and more than twice the risk of suicidal planning and attempts^23^. A meta-analysis of over 3.5 million participants found a 1.5-fold increased risk of chronic obstructive pulmonary disease among never smoking e-cigarette users^24^. These findings highlight ongoing uncertainty regarding the long-term risks of e-cigarette use among youth and young adults.

### Adolescents and nicotine dependence

Since 2017, e-cigarettes have become the most common first nicotine-containing product used by adolescents (12–17 years) in the United States^1,25^. More than two-thirds of teens who try any tobacco product begin with e-cigarettes^25^. The time to first cigarette after waking is considered a key clinical indicator of tobacco dependence^26^. Among teenagers, the proportion using e-cigarettes within 5 min of waking increased from under two percent to over ten percent, suggesting increasing levels of nicotine dependence^25^. Despite these trends, evidence supporting vaping cessation interventions in adolescents remains limited to a single behavioral RCT. Given the unique developmental and behavioral considerations in this population, additional trials evaluating targeted cessation interventions among adolescents are needed.

### Dual users of e-cigarettes and conventional cigarettes

Many individuals who attempt to switch from conventional smoking to e-cigarettes as a safer alternative often become dual users, a pattern associated with the highest health risks. A 2024 meta-analysis^27^ synthesized data from 107 studies. The authors reported that dual use was associated with increased risk of asthma, chronic obstructive pulmonary disease (COPD), stroke, metabolic dysfunction, and oral disease when compared to exclusive cigarette smoking. Compared to no nicotine product use, dual use was linked to higher odds of cardiovascular disease, stroke, metabolic dysfunction, asthma, COPD, and oral disease^27^. Flacco et al.^28^ reported a higher 6 year prevalence of smoking-related disorders among dual users compared to exclusive smokers. There is a risk that individuals attempting to quit vaping may transition to dual use, which significantly increases health risks. These findings further support the importance of developing effective vaping cessation interventions to prevent dual use and reduce associated harm.

### Biochemical validation in trials

Biochemical verification is essential for maintaining accuracy and scientific rigor in vaping cessation trials, yet fewer than half of the reviewed trials incorporated it into their study design. As highlighted by Benowitz et al.^29^, reliance solely on self-reported abstinence can lead to significant misclassification, with studies showing that approximately one out of every nine individuals who report quitting fail biochemical validation. This issue is particularly pronounced in populations prone to underreporting – such as adolescents^30^, female individuals^31^, and hospitalized patients^32^. Furthermore, quit rates may be overestimated by as much as 50% when relying on self-reports alone. Exhaled carbon monoxide measurement, primarily a byproduct of combustion, has limited utility for verifying vaping abstinence as standard e-cigarette use produces substantially less carbon monoxide than combustible tobacco^29^. Cotinine, a major nicotine metabolite detected in serum, urine, and saliva, has a prolonged half-life, providing a five to 7 day detection window for assessing nicotine exposure^33^. Cotinine can provide objective evidence of nicotine exposure among e-cigarette users, while additional biomarkers (e.g. NNAL, carbon monoxide, cyanoethyl mercapturic acid-to-cotinine ratio) can help differentiate between e-cigarette and combustible tobacco use^29^. Despite added costs and logistical challenges, incorporating biochemical verification enhances validity and helps avoid inflated efficacy claims in cessation trials.

### Financial incentives and their role in cessation outcomes

Most of the reviewed RCTs provided participant compensation to promote retention and optimize follow-up rates, raising concerns about the generalizability of study outcomes. A Cochrane review on incentives for smoking cessation, encompassing 33 mixed-population studies (n=21600 participants), provided high-certainty evidence that incentives improve smoking cessation rates at long-term follow-up^34^. In 2024, Kendzor et al.^35^ reported results from an RCT in which participants (n=320 participants) with low socioeconomic status were randomized to either usual care (counseling plus pharmacotherapy) or usual care combined with abstinence-contingent financial incentives^35^. No significant differences were observed in rates of 7 day PPA, 30 day PPA, or continuous abstinence at 26 weeks^35^. Instead, compensation was likely effective in improving recruitment and retention rates, which are critical for the robustness of trial findings^36^.

### Future research

Future research should focus on developing targeted interventions for high-risk populations, including adolescents, gender minorities, and racially and ethnically diverse groups. Interventions specifically designed for dual users may address their unique patterns, motivations, and barriers to quitting. Optimizing pharmacotherapy requires comparing different dosing strategies and extended treatment durations beyond 12 weeks. Vape-tapering strategies may be further explored to identify optimal protocols and integration with behavioral counseling or pharmacotherapy. Advancing digital cessation tools, such as mobile apps, chatbots, and text-based programs, may enhance effectiveness through AI-driven personalization, gamification (e.g. competition with others, point scoring), and real-time feedback. Finally, financial incentives may be incorporated not only to promote cessation but also to improve biochemical verification rates, participant retention, and the accuracy of self-reported abstinence^36^.

### Limitations

This review has several potential limitations. As a narrative review, the study did not follow formal systematic review procedures recommended by reporting guidelines, nor did it include pooled effect estimates or formal meta-analytic comparisons between interventions. In addition, the available evidence base remains limited by small sample sizes, heterogeneous study designs, variable outcome definitions, and generally short follow-up durations across the included RCTs. Finally, reliance on published studies may introduce publication bias.

## Conclusion

Current evidence suggests that behavioral interventions and varenicline may support vaping cessation, particularly among adolescents and young adults, although the available RCTs remain limited by small sample sizes and short follow-up durations. With millions developing nicotine dependence through e-cigarettes and minimal data on their long-term safety, stronger evidence is needed to develop vaping cessation strategies. Larger, well-powered trials are necessary to evaluate tailored behavioral interventions and optimize pharmacological treatment protocols for sustained vaping cessation.
